# Natural products proposed for the management of Huntington’s disease (HD): a comprehensive review

**DOI:** 10.1007/s00210-025-04444-w

**Published:** 2025-07-17

**Authors:** Aya Salman, Aya H. Eid, Samar S. Khalaf, Ahmed M. El-Dessouki, Riham A. El-Shiekh, Shaza H. Aly

**Affiliations:** 1 Department of Biochemistry, Faculty of Pharmacy, Egyptian Russian University, Badr City , Cairo, 11829 Egypt; 2 Pharmacology and Toxicology Department, Faculty of Pharmacy, Heliopolis University, Cairo, Egypt; 3 Biochemistry Department, Faculty of Pharmacy, Heliopolis University, Cairo, Egypt; 4 Pharmacology and Toxicology Department, Faculty of Pharmacy6 of October City, Ahram Canadian University, Giza, 12566 Egypt; 5 Department of Pharmacognosy, Faculty of Pharmacy, Cairo University, Kasr El-Aini Street, Cairo, 11562 Egypt; 6 Department of Pharmacognosy, Faculty of Pharmacy, Badr University in Cairo (BUC), Cairo, 11829 Egypt

**Keywords:** Huntington’s disease, Herbal medicines, Natural compounds, Neurodegenerative

## Abstract

Huntington’s disease (HD), a neurodegenerative disease, typically begins in the prime of adulthood, followed by a gradual onset of specific mental abnormalities and cognitive and physical impairment. To the best of our knowledge, no medication exists to totally stop the progression of HD. Among numerous therapy techniques, extensive literature reviews have confirmed the medicinal importance of natural products in HD experimental models. This review provides a literature survey of natural compounds and medicinal plants used as neuroprotective agents against HD. Relevant studies were found in a variety of scientific databases, including PubMed, ScienceDirect, Scopus, and Google Scholar. Overall, natural products provided various levels of neuroprotection in preclinical HD investigations through antioxidant and anti-inflammatory activities, mitochondrial function maintenance, apoptosis suppression, and autophagy induction. Plants such as *Bacopa monnieri*, *Ginkgo biloba*, *Panax ginseng*, and *Withaniasomnifera* were identified as the most promising anti-HD possibilities, with several of them known as CNS-active medicines. Curcumin, epigallocatechin-gallate, ginsenosides, kaempferol, naringin, and resveratrol were identified as anti-HD compounds, some of which are well recognized neuroprotectants. Further study is required to assess the therapeutic efficacy of new herbal extracts in HD animals.

## Introduction

Huntington’s disease (HD) is a genetically inherited autosomal-dominant neurodegenerative disorder that affects muscle coordination and causes slow nerve cell death, chorea and dystonia, motor ataxia, cognitive decline, and psychiatric issues (Kumar and Garg 2016). The majority of affected people show symptoms in their mid-adult years (35–45), but the condition can begin earlier in life. Chorea is an abnormal jerky, involuntary writhing movement induced by the disease (Nucifora 2001). Suicide rates among HD patients and carers were also found to be higher than in the general population. This disparity was attributed to reasoned suicide unrelated to mental illness (Bachoud-Lévi et al. 2019a). Today, two decades after the genetics of HD were found, pathogenesis remains poorly understood, and no effective treatment has been discovered. There are just a few therapeutic options for the physical, mental, and cognitive aspects of HD (Dickey and La Spada 2018). Tetrabenazine, the only medication for chorea, is also known to have limitations such as drug interactions and adverse effects (Setter et al. 2009; Jankovic and Clarence-Smith 2011). Selective serotonin reuptake inhibitors (SSRIs) and mood stabilizers have also been shown to be effective in treating early HD symptoms (Jimenez-Shahed and Jankovic 2013). Medicinal plants have historically provided key leads against a variety of ailments, including neurogenerative disorders (Dey and De 2015; Ratheesh et al. 2017; Yu and Bega 2019; Banerjee et al. 2022). This review addressed the recent research of plant extracts and isolated compounds that have impacts on neurotoxic and transgenic HD models.

## Etiology

HD is a dominantly hereditary condition marked by the impairment and demise of cortical and striatal neurons (Ratié and Humbert 2024). HD results from the extension of a polyglutamine coding (CAG) repeat at 4p16.3 of the huntingtin (HTT) gene (MACDONALD et al. 1993; Colpo et al. 2017). Finding an elevated CAG repeat length in the HTT gene in a patient exhibiting the condition’s clinical symptoms is often how the diagnosis is verified (Bates et al. 2015; Stoker et al. 2022). Both heterozygous and the uncommon homozygous HD patients exhibit comparable ages of onset due to the pure, dominant behavior of the polyglutamine repeat expansion process; there is no further impact on onset age when a second enlarged polyglutamine HTT allele is added to the homozygotes (Lee et al. 2012).

The length distribution of the polymorphic exon 1 polyglutamine-coding repeat differs among ethnic groups. The usual range is defined as repeat lengths of 26 or less. Repeats 27–35 are considered intermediate and can spread into the disease-causing range upon transmission. HD may develop if a repeat length of 36–39 is inherited, but this is not always the case (lower penetrance alleles). HD symptoms appear when repeat lengths of 40 CAG units or more are inherited because they are nearly entirely penetrant (Bates et al. 2015; Jiang et al. 2023).

## Symptoms

The autosomal-dominant, progressive hereditary neurodegenerative condition known as HD is typified by the selective death of neurons in the cortex and striatum, which results in increasing motor dysfunction, cognitive decline, and neuropsychiatric symptoms and behavioral abnormalities (Colpo et al. 2017; Stoker et al. 2022). Neuropsychiatric manifestations, such as depression, apathy, and irritability, may emerge several years prior to the onset of motor symptoms (Craufurd et al. 2001; Mühlbäck et al. 2024).

Depressive symptomatology (DS) is the most common mental health symptom among HD patients which affects between 30 and 70% of people(Jellinger 2024). All stages of HD are characterized by depressive symptoms, which start in pre-symptomatic HD gene carriers and are closely linked to suicidal thoughts and actions (Jellinger 2024). Several neuropathological mechanisms have been hypothesized to explain the development of DS in HD. These mechanisms include an early loss of neurons in the medial caudate, which is connected to limbic regions (Vonsattel et al. 1985b; Peyser and Folstein 1990). Depression, however, could just as easily result from other causes, such as a psychological response to being at risk for HD, having lived in a dangerous and insecure environment, and/or being aware of the disease’s start (Gamez et al. 2014).

Apathy is the most prevalent psychological manifestation of HD (Soliveri et al. 2022). Once present, it tends to worsen or remain, with the highest percentage occurring in the latter stages of the disease (van Duijn et al. 2014). A defining feature of apathy is less goal-directed conduct, marked by a decreased motivation to participate in cognitively or physically demanding activities, passive behavior, and a loss of interest (Ghosh and Tabrizi 2015; Atkins et al. 2024). It is regarded as an inherent characteristic of HD, evident even in the prodromal stage of the illness, correlates with disease advancement, serves as an indicator of functional deterioration, and has a major influence on quality of life (Martinez-Horta et al. 2016). Apathy exhibits the most robust linear correlation with illness progression, signifying a direct relationship with increasing neurodegeneration (Tabrizi et al. 2013; van Duijn et al. 2014). Consequently, apathy is generally associated with numerous other deficits, including involuntary motions and impaired sensory processing (van Duijn et al. 2010). Due to common characteristics and frequent overlap, it can be challenging to differentiate between depression and apathy (Connors et al. 2023).

One other common neuropsychiatric sign of HD is irritability; it is a prevalent and debilitating issue linked to HD (Nimmagadda et al. 2011; Reedeker et al. 2012). Since irritability has nothing to do with HD’s motor or cognitive characteristics, it is a distinct neuropsychiatric symptom of the disease (Nimmagadda et al. 2011). Irritability is usually referred to as the initial indication of HD in pre-symptomatic patients, but it can happen at any point in the course of the illness, though it is more common in those whose neurological symptoms have persisted for 6 to 11 years (van Duijn et al. 2008; Smith et al. 2012). The intricate link between the basic neurobiological alterations brought on by the disease progression and the secondary psychological impacts, which are a response to the subjective changes patients undergo, is believed to be the cause of irritability in the setting of HD (Simpson et al. 2019). An example is the progressive cognitive deterioration brought on by the condition, which can be perceived as a form of cognitive overload and lead to irritation. This is how it differs from irritation in the general population (McCusker and Loy 2014). Irritability is identified using standardized rating scales and clinical knowledge. Many factors make it difficult to make a precise and conclusive diagnosis, including determining its severity and progression. These include the co-occurrence of other neuropsychiatric symptoms with irritability, the unique features of irritability in HD that are not exactly comparable to the symptom in the general population, and the cognitive challenges or lack of insight HD patients may experience (Mestre et al. 2016).

On the other hand, involuntary movements (such as chorea, stiffness, and dystonia) and impairments to voluntary movements (such as trouble organizing and finishing tasks, dysarthria, dysphagia, and akinesia) are characteristics of motor dysfunction (Peball et al. 2022; Gunn et al. 2023). While deterioration in executive decision-making processes, especially in goal-oriented behaviors, is a sign of cognitive flaws. Verbal learning impairment and visuospatial problems are frequent, and finally, extensive memory impairment becomes apparent (Hergert and Cimino 2021; Horta-Barba et al. 2021). Moreover, the cerebral cortex is one of the other brain regions that deteriorate as the illness worsens. Changes are also observed outside of the brain, specifically in the areas of cachexia and weight loss (Liot et al. 2017).

In addition, patients with HD frequently report experiencing sleep disturbances, which can be extremely distressing and are seen from the beginning of the illness or even before clinical symptoms appear (Videnovic et al. 2009; Herzog-Krzywoszanska and Krzywoszanski 2019; Ogilvie et al. 2021). The most common sleep issues that HD patients report include excessive daytime sleepiness, insomnia, trouble falling asleep, and frequent nocturnal awakenings (Herzog-Krzywoszanska and Krzywoszanski 2019).

## Diagnosis

HD is typically diagnosed by combining genetic testing (polymerase chain reaction) with the patient’s family history and clinical signs; clinical evaluations such as the observation of motor symptoms, mental health issues, and cognitive deterioration have been used to diagnose HD (McColgan and Tabrizi 2018; Stoker et al. 2022). Pre-symptomatic, prodromal, and manifest HD are the three categories into which current classification techniques divide HD (Ross et al. 2019). Pre-symptomatic HD refers to people who have the CAG expansion but do not yet exhibit any HD-related symptoms. People with the CAG expansion who have mild but noticeable cognitive problems and nonspecific or potential motor abnormalities on examination are considered to have prodromal HD. Manifest HD comprises people with the CAG expansion who have a larger than 90% likelihood of motor abnormalities together with modest or significant neurocognitive changes, or people who have a greater than 99% probability of motor abnormalities but no change in cognition (Jaini et al. 2020; Medina et al. 2022).

Since early diagnosis opens the door to prompt therapies that may delay the progression of this debilitating neurodegenerative disease, the significance of early detection in HD cannot be emphasized (Ganesh et al. 2023). But as the illness worsens, these symptoms become more noticeable, making early diagnosis difficult. To overcome this difficulty, developments in genetic testing have made it possible to directly identify the expansion of the CAG repeat in the HTT gene, offering an accurate diagnosis (Tabrizi et al. 2020). Since genetic testing is so reliable, it has emerged as the gold standard for diagnosing HD (Ahmed and Mridha 2023). Individuals suspected of having HD without genetic testing are classified as at risk, clinically prodromal, or clinically present (Medina et al. 2022).

Even though certain imaging modalities, including diffusion-weighted magnetic resonance imaging (MRI), structured MRI, functional MRI, and fluorodeoxyglucose-positron emission tomography scan (FDG PET) scans, can identify the disease up to 25 years before it manifests, imaging is still infrequently employed today as they are costly and patients’ involuntary movements may degrade the image quality (McColgan and Tabrizi 2018; Stoker et al. 2022; Zhang et al. 2023). Another method, called magnetic resonance spectroscopy (MRS), has been utilized to measure metabolic changes in the brain by applying spectroscopic analysis of the MRI signal. HD patients have also been treated with this method (Zhu and Barker 2011). MRS can be a valuable tool for the early identification of HD; putative objective biomarkers associated with its start and pathophysiology exist and demonstrate variations between pre-HD and HD patients (Lozada et al. 2024).

Moreover, biofluid biomarkers produced mostly from CSF and blood can directly represent disease pathogenic processes at the molecular level and may uncover the possible pathogenesis of HD (Tabrizi et al. 2020; Niemela et al. 2021). Furthermore, assays and blood-based biomarkers that quantify particular proteins or metabolic alterations are being researched for their potential as diagnostic tools (Baskota et al. 2019). The blood-based biomarkers are intended to immediately identify the peripheral pathophysiological alterations of HD or, ideally, to partially mirror the concurrent central neuropathogenic processes (Zhang et al. 2023). There is potential for improving early and precise HD diagnosis by combining several biomarkers with clinical evaluations and genetic testing.

## Incidence and prevalence

The frequency and prevalence of HD have been clarified by recent epidemiological research, exposing the disease’s harsh reality. Globally, there are approximately 3.92 cases of HD for every 100,000 people (D’Egidio et al. 2024). According to reports from different populations, the estimated pooled incidence of HD new cases is 0.48 cases per 100,000 person per year (95% CI, 0.33–0.63). Regional differences in HD prevalence are more than ten times greater. This discrepancy can be attributable in part to changes in case determination and/or diagnostic criteria (Rawlins et al. 2016). The prevalence of HD was substantially higher in Europe and North America than in Africa in a previous study. Also, the prevalence of HD was considerably greater in Europe and North America than in East Asia, if Asia is further separated into the Middle East and East Asia (0.41 per 100,000; 95% CI, 0.36–0.47) (Rawlins et al. 2016; Medina et al. 2022). Due to its rarity, identifying HD, especially early on, is difficult. In addition to incidence, understanding HD prevalence is crucial for efficient resource allocation and healthcare planning. The combined prevalence of HD is 4.88 (95% CI: 3.38–7.06) per 100,000 (Medina et al. 2022; D’Egidio et al. 2024). The entire burden of HD within populations is revealed by the collected prevalence, which is 4.88 per 100,000 (95% CI, 3.38–7.06) (Medina et al. 2022; Ganesh et al. 2023).

## Risk factors

### Genetic mutation

The most significant contributing element to the development of HD seems to be genetic (Chao et al. 2017). The length of the HTT CAG repeat expansion, which is the main factor influencing the rate at which the disease develops leading to disease onset, is the only factor contributing to the genetic risk of HD (Lee et al. 2015). It was discovered that the mutant allele’s CAG repeat length was a reasonably constant and important risk factor for the development of HD, particularly in the degradation of motor, cognitive, and other neurological symptoms (Chao et al. 2017). A greater number of CAG repeats was also linked to early percutaneous endoscopic gastrostomy, quicker institutionalization, and a lower survival time, according to numerous consistent findings in the literature (Chao et al. 2017). An elevated risk of the relevant psychiatric symptoms in HD is linked to polygenic risk scores for psychiatric disorders, including schizophrenia and depression, indicating a shared genetic vulnerability (Ellis et al. 2020).

### Family history

Overall illness symptoms and an earlier onset of depression seem to be linked to a positive family history of HD (Kringlen et al. 2017). A positive family history of HD is still a useful indicator for late-onset HD diagnosis (Koutsis et al. 2014). A sensitive and specific genetic test, along with an autosomal dominant family history, enables pre-clinical diagnosis many years before any usual clinical symptoms appear (Klöppel et al. 2009).

### Dietary intake

Patients who consume more calories have a higher likelihood of having more CAG repeats (> 37), a higher risk of developing the disease, and experience HD earlier (Marder et al. 2013). In individuals with advanced HD, a sufficient nutritional intake avoids weight loss, but it is not connected to improved functional condition (Cubo et al. 2015). Dietary fiber treatments may be able to postpone the onset of symptoms in HD and may also have consequences for other conditions where the gut-brain axis is disrupted (Gubert et al. 2024).

### Age

Age significantly affects the pathogenic hallmark of mHTT protein aggregation as the disease progresses (Bhat et al. 2024). Most of the time, aging speeds up HD due to the accumulation of cellular and molecular changes that exacerbate the harmful effects of mHTT (Lee et al. 2020). Moreover ageing leads to increased protein misfolding, mitochondrial dysfunction, and DNA damage in cells (Maity et al. 2022).

## Experimental models overview

**Fig. 1:**
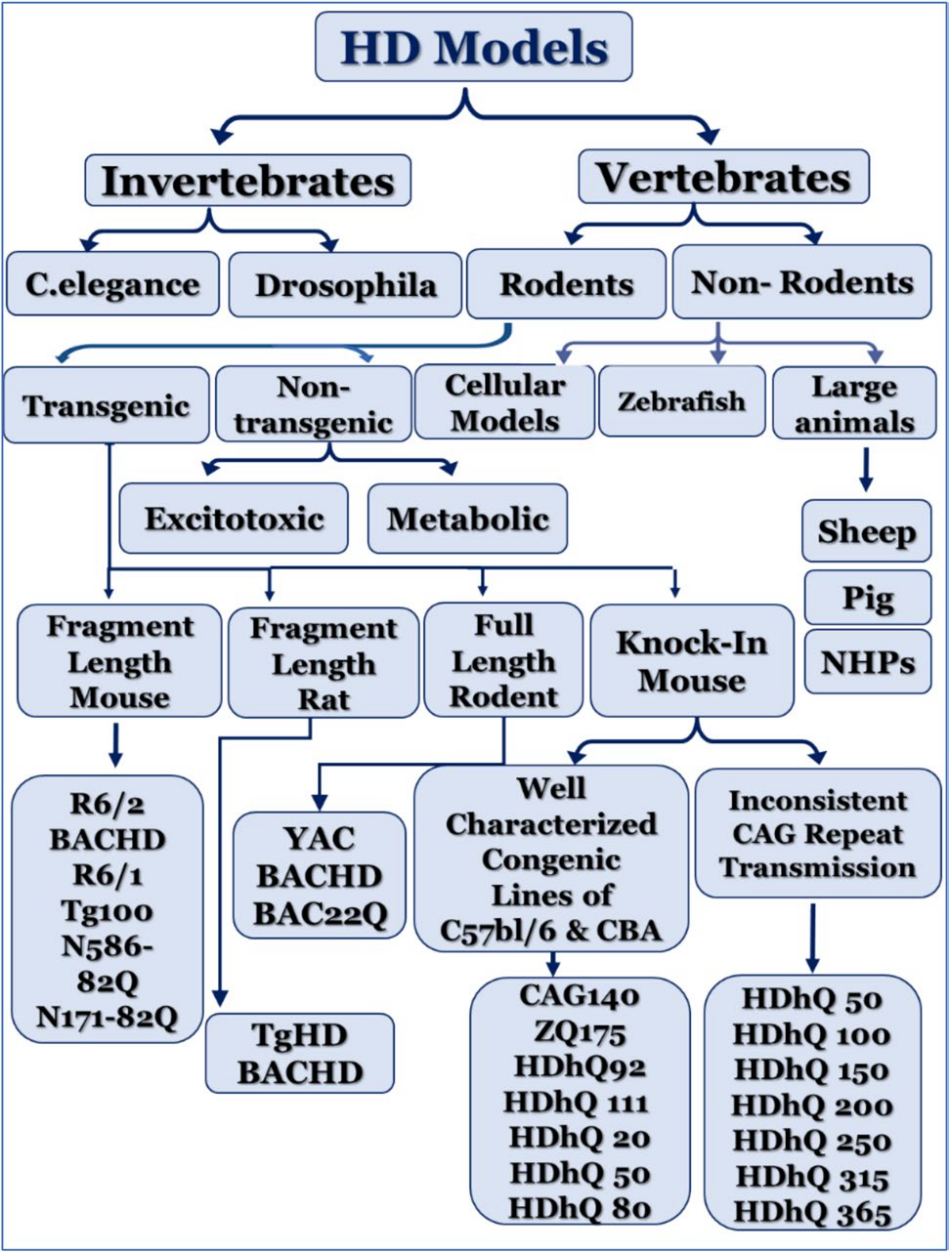
Schematic classification of the animal models developed for modelling HD

The goal of HD experimental models is to mimic the disease’s genetic, molecular, and behavioral features. These models include two main large categories: vertebrate-based models and invertebrate-based models. These two categories are divided into many subclasses as shown in (Fig. 1).

### Invertebrate animal models

#### Caenorhabditis elegans model

The nematode *C*. *elegans* is a transparent kind of worm used to study Huntington’s disease (HD) via transgenic polyQ expression, enabling real-time tracking of neurodegeneration and neuronal dysfunction (Faber et al. 1999; Brignull et al. 2006; Gonzalez-Moragas et al. 2015). Its genetic tractability, optical accessibility, and high human gene homology (~ 60–80%) make it valuable for mechanistic studies (Gonzalez-Moragas et al. 2015; Rieckher and Tavernarakis 2017; Roussos et al. 2023; Yu et al. 2024). However, its simplified nervous system and lack of HTT ortholog limit clinical translatability (Yu et al. 2024).

### Drosophila melanogaster

The Drosophila HD model expresses expanded polyQ repeats in photoreceptors, causing progressive neurodegeneration with visible inclusion formation (Jackson et al. 1998). Its short lifespan and genetic tractability enable high-throughput studies (Ramaswamy et al. 2007).

#### Vertebrate animal models

### Toxin-induced models

## Quinolinic acid (QA) model

Quinolinic acid selectively targets medium spiny neurons (MSNs) in the striatum, mimicking HD’s excitotoxic mechanisms (Ca^2^⁺ dysregulation, ATP depletion) and manifesting an early-stage hyperkinetic symptoms (Bordelon et al. 1997, Gárdián and Vécsei 2004; Nittari et al. 2023). It upregulates HTT protein expression and shows cognitive deficits in Morris water maze tests (Shear et al. 1998). Limitations include acute progression (vs. HD’s chronic course) and inability to model late-stage hypoactivity (Roberts et al. 1993; Vazey et al. 2006).

## 3-nitropropionic acid (3-NP) model

3-Nitropropionic acid inhibits mitochondrial complex II, causing striatal degeneration resembling HD pathology (Palfi et al. 2000). Dose-dependent effects replicate hyperkinetic (early HD) or hypokinetic (late HD) symptoms (Brouillet et al. 1995; Borlongan et al. 1997). The object retrieval detour task (ORDT), which intentionally strains working memory and depends on intact frontostriatal circuitry, shows cognitive abnormalities in monkeys treated with 3-NP (Palfi et al. 2000). Strain variability (Fischer > Lewis rats) and selective dorsolateral striatal vulnerability are noted (Vonsattel et al. 1985a; Ouary et al. 2000; Blum et al. 2001).

## Genetic models of Huntington’s disease

### Transgenic models

Transgenic HD models, including R6/2, N171-82Q, and full-length YAC/BAC mice, express mutant huntingtin (HTT) with expanded CAG repeats, recapitulating key pathological and behavioral features of the disease. The R6/2 mouse, expressing exon 1 of human HTT with ~ 144 CAG repeats, exhibits rapid progression (onset at 4–11 weeks) with severe motor deficits, cognitive impairments, and widespread neuronal inclusions, making it valuable for juvenile HD studies (Sun et al. 2002; Stack et al. 2005). The N171-82Q model, expressing a 171-amino acid N-terminal fragment with 82 CAG repeats, shows slower progression (symptoms at 11–16 weeks), striatal neurodegeneration, and better modeling for adult-onset HD (McBride et al. 2004). Full-length models such as YAC128 and BACHD express full-length mutant HTT (128 and 97 CAG repeats, respectively) display progressive motor/cognitive decline, striatal atrophy, and synaptic dysfunction, closely resembling human HD progression (Slow et al. 2003; Petersén et al. 2005; Van Raamsdonk et al. 2005).

### Knock-in (KI) models

Knock-in models, including CAG140 and zQ175, integrate expanded CAG repeats into the endogenous mouse HTT locus, providing genetic fidelity and gradual disease progression. These models develop late-onset motor deficits (e.g., rotarod impairments at 9–12 months) and nuclear HTT aggregates but often lack overt neuronal loss, limiting their utility for studying advanced pathology (Menalled 2005; Stricker-Shaver et al. 2018). The CAG150 KI model shows more severe phenotypes, including striatal gliosis and axonal degeneration, suggesting that higher CAG repeats may better replicate human HD (Lin et al. 2001; Yu et al. 2003).

### Non-rodent models

#### Sheep models

The OVT73 transgenic sheep model expresses mutant HTT at low levels, making it particularly useful for studying early-stage HD pathology. These animals exhibit white matter alterations in the corpus callosum and metabolic disturbances similar to human HD patients, though they lack overt motor symptoms before age 5 (Taghian et al. 2022; Morton 2023; Spick et al. 2023). Their large brain size and longevity facilitate long-term therapeutic studies, while advanced imaging techniques reveal progressive neurological changes (Morton et al. 2014; Mears et al. 2021). However, the absence of validated cognitive tests limits behavioral assessments in this model (Morton and Avanzo 2011).

#### Non-human primate (NHP) models

Non-human primate models provide the closest neuroanatomical and behavioral parallels to humans, with transgenic models displaying key HD features such as dystonia, chorea, and cognitive decline (Guo et al. 2018; Yan et al. 2019). The first NHP HD model, created using a lentiviral vector carrying exon 1 of human HTT with 84 CAG repeats, showed severe neuronal toxicity and aggregation (Yang et al. 2008). A more refined model expressing 508 amino acids of HTT with 67–72 CAG repeats better replicates chronic HD progression (Chan et al. 2015). Despite their translational value, ethical concerns, high costs, and long reproductive cycles hinder widespread use (Coe and Lubach 2021; Tian 2021).

#### Pig models

Pigs offer a balance between neuroanatomical similarity to humans and practical advantages over NHPs, including shorter gestation periods and larger litter sizes (Pirone et al. 2023). Early transgenic pig models expressing N-terminal HTT fragments (N208-105Q) showed abundant aggregates but lacked behavioral phenotypes (Yang et al. 2010). CRISPR-edited knock-in pigs with 150 CAG repeats exhibit progressive motor deficits, striatal neurodegeneration, and germline transmission of HD pathology, closely mirroring human disease progression (Ran et al. 2013; Yan et al. 2018). These models are increasingly valuable for preclinical testing due to their physiological relevance and scalability.

### Cellular models

#### Organoids and iPSC-based models

iPSC/Organoid Models iPSC-derived neurons and brain organoids with mutant HTT reveal developmental defects, including impaired neural maturation and disrupted cortical organization. These human cell models enable mechanistic studies and drug screening in patient-specific neurons (Pardo Muñoz et al. 2017; Conforti et al. 2018; Faravelli et al. 2020).

### Zebrafish models of HD

Zebrafish provide a cost-effective system for studying HTT function, showing conserved roles in neurodevelopment and iron metabolism. Their transparent embryos allow real-time observation of neural defects, though they lack the extended polyQ repeats of human HD (Karlovich et al. 1998) (Rink and Wullimann 2001; Lumsden et al. 2007; Henshall et al. 2009; Lo Sardo et al. 2012).

## Drugs used in the management of chorea in HD

**Fig. 2:**
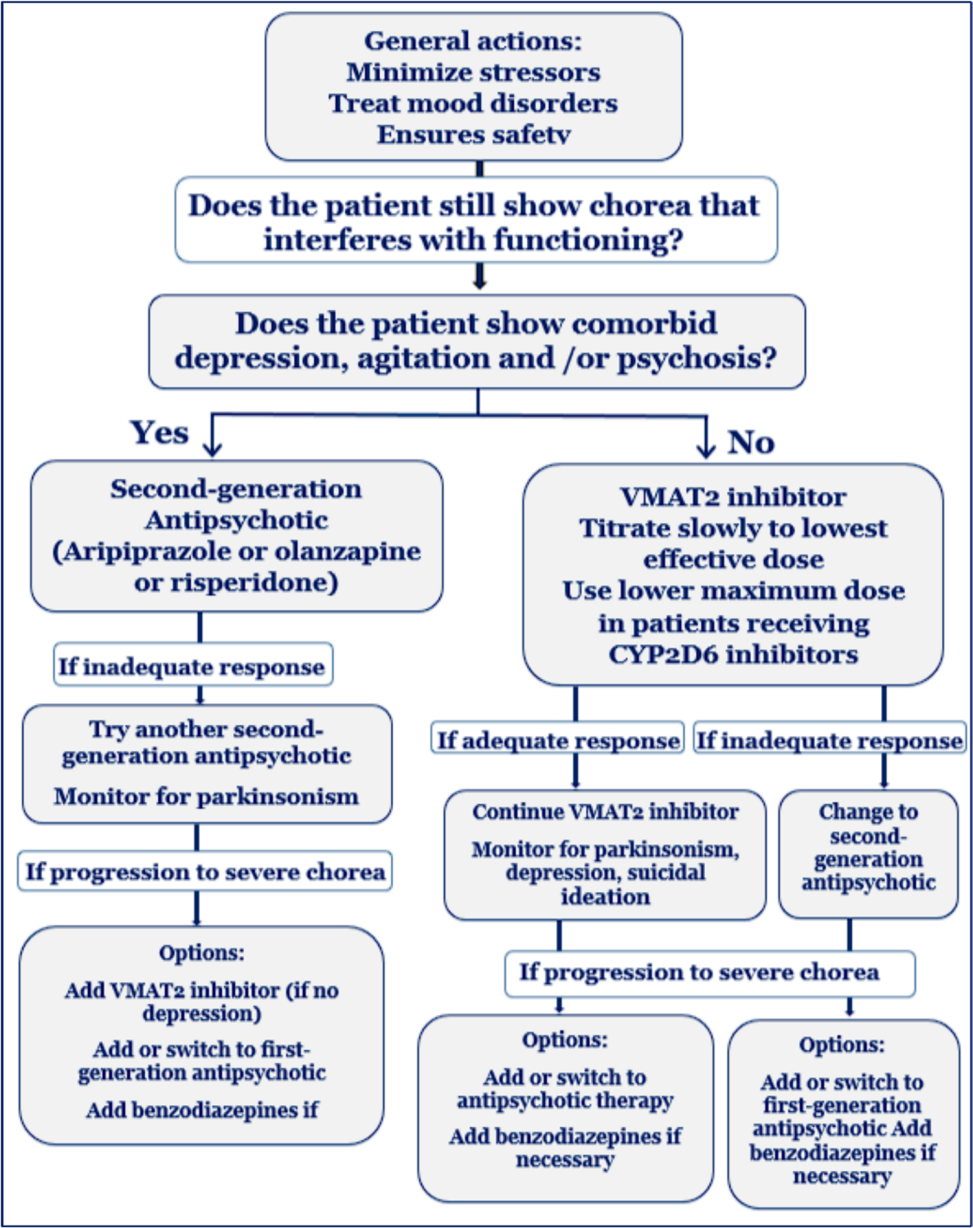
Treatment algorithm for chorea in HD (Furtado and Suchowersky 1995)

Only in situations where chorea interferes with the patient’s ability to function, a pharmacological therapeutic approach is indicated (Armstrong and Miyasaki 2012). While reducing this symptom can improve speech and rest, as well as lower the chance of falls or choking, the drugs used may cause depression, suicidal thoughts, and parkinsonism. Therefore, a thorough risk–benefit analysis must always be the foundation for the choice to begin treatment. This article focuses on the pharmacogenetic information of the medications used in accordance with the HD treatment algorithm for chorea as shown in (Fig. 2) (Furtado and Suchowersky 1995).

### Monoamine transporter type 2 (VMAT2) inhibitors

In general, vesicular monoamine transporter type 2 (VMAT2) inhibitors are recommended for the treatment of chorea. In 2017, the FDA approved Deutetrabenazine (Austedo®), a deuterated derivative of Tetrabenazine, which was the first approved agent. Deutetrabenazine’s slower metabolism results from substituting deuterium for hydrogen at six locations, which lessens the requirement for frequent doses (Schneider et al. 2021). However, Deutetrabenazine is currently not commercialized in Europe and other countries. In addition to the potential risk of suicidal episodes, QT prolongation, and neuroleptic malignant syndrome, frequent adverse effects such as drowsiness, sedation, depression, akathisia, parkinsonism, and extrapyramidal symptoms could also potentially happen (Frank et al. 2022).

The metabolites α-dihydrotetrabenazine (α-HTBZ) and β-dihydrotetrabenazine (β-HTBZ), which selectively bind VMAT2, inhibit monoamine transport, and deplete dopamine and other neurotransmitters in the CNS, are produced quickly by carbonyl reductase from both tetrabenazine and deutetrabenazine. Tetrabenazine is prescribed at a starting dose of 25 mg three times a day. Depending on tolerance, this dosage may be increased by an additional 25 mg every 3 to 4 days, up to a maximum of 200 mg per day. In the USA, deutetrabenazine is authorized to treat tardive dyskinesia and chorea associated with HD. Starting with 6 mg once daily, the suggested dosage can be increased by 6 mg per week until it reaches a maximum of 48 mg daily (24 mg twice daily). But for patients who are concomitantly using a strong CYP2D6 inhibitor such as quinidine, paroxetine, fluoxetine, or bupropion or having the CYP2D6 poor metabolizer phenotype should not take more than 36 mg per day, with a maximum of 18 mg each dosage (García-González et al. 2023).

### Second-generation antipsychotic drugs for management of chorea

In individuals for whom VMAT2 inhibitors are contraindicated, such as those with depression, or when VMAT2 inhibitors are unable to adequately control symptoms, antipsychotic drugs are frequently recommended for chorea (Brusa et al. 2009). HD-related psychological symptoms can also be treated with these drugs. Second-generation antipsychotics mainly work by blocking D2 dopamine receptors and serotonin receptors, especially 5-HT2A. Because they are less likely to have extrapyramidal adverse effects, they are typically chosen over first-generation medications. The CYP2D6 enzyme plays a major role in the metabolism of risperidone and aripiprazole (Gupta and Masand 2004).

Patients who exhibited the CYP2D6 poor metabolizer (PM) phenotype were advised to modify their dosage by both European and U.S. regulatory agencies. A 50% dose reduction is recommended for oral aripiprazole, and in patients with CYP2D6 PM, the initial and maintenance doses of the intramuscular extended-release formulation should be 300 mg instead of 400 mg; if strong CYP3A4 inhibitors are also being taken at the same time, the dose should be further reduced to 200 mg (Garcia-Gonzalez et al. 2023). In patients with the CYP2D6 PM phenotype, the Dutch Pharmacogenetics Working Group (DPWG) recommends lowering the maximal dosage of aripiprazole to 68–75% (Swen et al. 2011).

Although there are no particular pharmacogenetic guidelines or recommendations for olanzapine, patients taking CYP1A2 inhibitors, such as fluvoxamine, are advised to start with a lower dose because CYP1A2 is essential for its metabolism. Furthermore, individuals with schizophrenia who are receiving treatment with first- and second-generation antipsychotics may gain weight due to genes like leptin (LEP) and cannabinoid receptor 1 (CNR1) (Tiwari et al. 2010). Short-term treatment with aripiprazole and olanzapine had a significant impact on metabolic parameters, including higher levels of prolactin, C-peptide, glucose, and insulin (Koller et al. 2021).

### First-generation antipsychotic drugs

Considering the greater potency of first-generation antipsychotics, they are usually used in situations of severe chorea refractory to VMAT inhibitors or second-generation antipsychotics. However, there is a higher chance of side effects such as akathisia, parkinsonism, sedation, dystonia, and hypotension with these drugs. Haloperidol is one of the most often given of these medications, with daily dosages usually ranging from 0.5 mg to 10 mg (Unti et al. 2017) which is mostly metabolized by UGT2B7, UGT1A9, and UGT1A4 by glucuronidation (50–60%) (Kato et al. 2012).

### Other drugs used in the management of chorea

Although long-term use of benzodiazepines, such as clonazepam and lorazepam, is typically discouraged, they can be used temporarily to relieve severe chorea episodes. A number of benzodiazepines are mainly or partially metabolized by the polymorphism-exhibiting enzymes CYP2C19 and CYP3A4/5. It has been demonstrated that the polymorphism in CYP2C19 specifically affects the pharmacokinetics of desmethylclobazam, quazepam, etizolam, and diazepam (Fukasawa et al. 2007).

CYP3A4 plays a major role in the metabolism of midazolam, clonazepam, alprazolam, and triazolam. Patients receiving clonazepam, who are poor metabolizers (PMs) should be started with a daily dose of 5 mg and titrated progressively based on weight [64]. Additional medications being investigated for the management of chorea including amantadine, cannabinoids (cannabidiol, nabilone), and anticonvulsants like levetiracetam and topiramate are exhibiting promises; yet, there is still little supporting data (Garcia-Gonzalez et al. 2023).

## Pharmacogenetics of the drugs used in the management of comorbidities of HD

Between 33 and 76% of patients with HD are anticipated to have psychological problems at some point in their lives. Surprisingly, 98% of HD patients who experience motor symptoms are expected to experience at least one psychological symptom as well (Paulsen et al. 2001). Notably, depression is more common in pre-symptomatic HD gene carriers and can manifest up to 10 years prior to the onset of motor symptoms (Duff et al. 2007). The most incapacitating feature of HD is usually not the physical deficits but rather these psychosocial symptoms, which are strong predictors of the requirement for long-term care and often result in hospitalization (Loi et al. 2018).

### Depression and sleep disturbances

Depression is one of the most common mental disorders observed in HD which significantly impacts patients’ quality of life. Cognitive-behavioral therapy and psychotherapy may be able to help identify mood swings early. Antidepressant medication may be necessary if patients have moderate (grade B) depression (Beglinger et al. 2014). Usually, serotonin-norepinephrine reuptake inhibitors (SNRIs) or selective serotonin reuptake inhibitors (SSRIs) are advised. Alternative drugs like mirtazapine may be taken into consideration when sleep problems are evident (Bachoud-Lévi et al. 2019b). Guidelines for SSRIs are provided by the Clinical Pharmacogenetics Implementation Consortium (CPIC), which emphasizes the significance of modifying dosages according to the CYP2D6 and CYP2C19 genotypes, especially for medications like sertraline, citalopram, escitalopram, fluvoxamine, and paroxetine. Dosing guidelines for paroxetine and fluvoxamine are based on CYP2D6 phenotypes and for citalopram, escitalopram, and sertraline based on CYP2C19 phenotypes (Hicks et al. 2015).

Additional investigations are warranted in order to clarify the efficacy of duloxetine in HD patients. For people with depression who also experience anxiety symptoms and sleep difficulties, mirtazapine can be beneficial (Anttila and Leinonen 2001). Options including venlafaxine, desvenlafaxine, and duloxetine may be taken into consideration when an SNRI is the recommended course of treatment. With careful monitoring of plasma metabolite levels, the DPWG recommends that patients with CYP2D6 PM or IM phenotypes either take a different medication or have their venlafaxine dosage decreased. It is advised that UMs either choose alternate therapy or raise the dosage by 150% (Swen et al. 2011).

### Anxiety

Patients with HD frequently experience anxiety, which is defined as uneasiness or concern about past or future events. The emotional toll of illness is frequently combined with financial, familial, and social difficulties. Additionally, anxiety is tightly linked to the decline of cognitive and motor abilities, which makes patients more anxious. Anxiety is also linked to depression, pain, irritability, and a poor quality of life. SSRIs or SNRIs are regarded as first-line therapies for anxiety, particularly when depression is present (Bachoud-Lévi et al. 2019b). Though they may be beneficial, on-demand anxiolytics should only be used sparingly because they may exacerbate the other symptoms. Neuroleptics can be used as a therapeutic alternative when conventional therapies are not successful. Furthermore, benzodiazepines can be used temporarily to treat agitation associated with anxiety; however, prolonged use of these drugs should be avoided to reduce any dependence hazards (Garcia-Gonzalez et al. 2023).

### Irritability and impulsivity

One of the hallmark symptoms of HD is irritability, which is frequently made worse by impulsivity. Irritability can result in loss of control and, in rare instances, aggressive behavior or even criminal actions. The first-line treatment for irritability is typically selective serotonin reuptake inhibitors (SSRIs), but achieving efficacy may necessitate using SSRIs at or close to their maximum recommended dosage (Bachoud-Lévi et al. 2019b).

### Apathy

Clinically, apathy is defined as a “quantitative loss in goal-directed behavior” (Levy and Czernecki 2006), which includes a decline in motivation, drive, interest, and spontaneity. It is the most prevalent behavioral and psychological symptom of HD, particularly in its middle and advanced phases, and it significantly impairs normal daily activities. The cognitive and psychological profiles of irritation and apathy frequently coincide (Bouwens et al. 2015). When depression coexists with apathy, the severity of the condition may worsen; in this scenario, SSRI use is advised. Additionally, since sedatives can make apathy worse, it is recommended to reduce their dosage or refrain from giving them excessively (Garcia-Gonzalez et al. 2023).

### Aggression

Neuroleptics are usually used as the first line of treatment when aggressiveness is a noticeable symptom. Haloperidol, SSRIs, and mirtazapine are the most frequently prescribed neuroleptics (Bachoud-Lévi et al. 2019b).

### Psychosis

Psychosis is a state in which a person loses perception of reality. This mental illness is accompanied by hallucinations and delusions. First-generation neuroleptics are usually used to treat these symptoms (Bachoud-Lévi et al. 2019b).

### Akinetic HD with Parkinsonian features

Clozapine is considered the drug of choice for people with severe Parkinsonian symptoms who have akinetic types of HD. A combination of atypical neuroleptics and serotonergic antidepressants may be beneficial in treating persistent ideation that mimics psychotic symptoms (Begum and Fathima 2020).

## Future directions for HD management

### ASOs: nucleicacid-based therapeutics

Recent clinical trials have explored antisense oligonucleotides (ASOs) for HD. Tominersen, a gapmer ASO developed by Ionis and Roche, demonstrated a 40% reduction in cerebrospinal fluid (CSF) mHTT levels in a phase 1/2a trial, with no severe adverse events (Leavitt 2024). However, in the GENERATION HD1 phase 3 trial, eight-weekly dosing showed worsened clinical outcomes and increased CSF neurofilament light (NFL) levels, raising concerns about tolerability. Post hoc analyses suggest younger patients with lower disease burdens may benefit from less frequent dosing (García-González et al. 2023).

Wave Life Sciences’ ASO trials, WVE-120101 and WVE-120102, aimed for allele-specific mHTT reduction via selective targeting of mutant HTT transcripts. Unfortunately, phase 1/2 trials reported no significant mHTT lowering in the CSF, indicating a lack of effective target engagement. These results underscore the complexities of ASO-based therapies for HD, highlighting the need for tailored dosing regimens and enhanced targeting strategies (Kingwell 2021).

## Gene therapy: advancements and challenges

Gene therapy employs transgenes encoding therapeutic molecules to combat and prevent diseases (High and Roncarolo 2019). However, efficient delivery of RNA interference (RNAi), zinc-finger proteins (ZFPs), and CRISPR-based genome editing tools to key regions of the central nervous system (CNS) remains a critical challenge. These unmodified constructs are unable to cross the blood–brain barrier (BBB) or be readily internalized by neurons. Currently, recombinant adeno-associated viral (AAV) vectors are the preferred method for achieving effective cellular transduction and stable transgene expression. AAV vectors offer several advantages, including being non-pathogenic, non-replicating in the host, and not integrating into the host genome (Miniarikova et al. 2018). Bilateral intraparenchymal infusion is commonly used to deliver these vectors to specific brain regions, with the striatum (caudate/putamen) being the primary target due to its severe involvement in HD. However, because HD affects the entire brain, broader delivery to cortical and other regions may be required for effective treatment. Despite their promise, AAV vectors have notable limitations. Immune responses to the viral vector often preclude repeated administration, and delivery is irreversible, preventing dose adjustment post-administration. Additionally, tissue distribution varies significantly, with transduction efficiency diminishing as the distance from the injection site increases. These challenges underscore the complexity of designing gene therapies for HD and the need for innovative solutions to improve delivery precision and overcome immune-related barriers (Vallès et al. 2021).

### Insights into AAV-mediated RNAi-based therapy

Preclinical investigations of adeno-associated virus (AAV)-mediated RNA interference (RNAi) therapies have demonstrated significant therapeutic potential for HD in transgenic animal models. Studies in HD mouse models revealed that a 40–60% reduction in HTT, encompassing both wild-type (wtHTT) and mutant HTT (mHTT), was well-tolerated, extended survival, and improved motor function even when initiated during symptomatic stages (Drouet et al. 2009). Similarly, in a transgenic HD minipig model, intracranial administration of AAV5-delivered anti-HTT microRNA (miRNA) resulted in extensive reduction of mHTT levels across multiple brain regions (Howland et al. 2020), with sustained suppression persisting for up to 1 year post-injection (Vallès et al. 2021). These findings laid the groundwork for the first human trial of AAV-mediated RNAi therapy, which reported a remarkable 45% reduction in HTT levels without inducing neuropathological abnormalities or adverse effects (McBride et al. 2008). Furthermore, long-term safety was corroborated by NHP studies, where total huntingtin lowering persisted without detectable toxicity for at least 6 months after administration. These encouraging outcomes underscore the translational potential of AAV-mediated RNAi therapy as a viable therapeutic strategy for HD (Grondin et al. 2012). Clinical trials for miRNA therapies in HD are progressing, including uniQure’s AMT-130 trial evaluating AAV5-miHTT delivery via MRI-guided intrastriatal injections and Spark Therapeutics’ planned trial for AAV1-delivered anti-HTT miRNA. Meanwhile, Voyager Therapeutics’ similar program is on hold pending improvements in viral delivery efficiency (McBride et al. 2008).

### CRISPR and ZEPs: DNA-targeted approaches

Therapeutic strategies targeting the HTT gene’s DNA hold transformative potential, as correcting the underlying genetic defect could eliminate all disease-associated pathogenicity. Zinc finger proteins (ZFPs) combine a DNA-binding domain of zinc finger peptides with an active effector, such as a transcriptional repressor or nuclease, allowing precise targeting of specific DNA sequences (Klug 2010). ZFPs have demonstrated the ability to selectively suppress mutant HTT (mHTT) expression while sparing wild-type HTT (wtHTT), with AAV-ZFP delivery reducing mHTT levels and improving HD-like behavioral phenotypes in mouse models (Zeitler et al. 2019). Similarly, CRISPR-based systems offer multiple, precise, and coherent technological platforms for gene editing in neurodegenerative diseases, including HD. CRISPR has successfully inactivated the mutant HTT gene in patient-derived fibroblasts (Shin et al. 2016), HD mouse models (Monteys et al. 2017), and human-induced pluripotent stem cells (Heman-Ackah et al. 2016). While preclinical evidence supports the feasibility of these convergences, no clinical trials have commenced. Advances in delivery systems, including viral and non-viral methods, and further validation in large animal models will be crucial before these DNA-targeting therapies transition to human studies (Tabrizi et al. 2022).

### Splicing modulators

Emerging therapies for HD include a novel class of oral, brain-penetrant small molecules designed to reduce HTT protein expression. Branaplam (LMI070), initially discovered through a phenotypic screen for spinal muscular atrophy (SMA), is undergoing phase II trials for SMA (NCT02268552) and has demonstrated the ability to decrease both mutant and wild-type HTT levels via splicing modulation. Its mechanism involves exon-49/50 mis-splicing, which triggers HTT mRNA decay and reduces protein production (Keller et al. 2022). Novartis received FDA Orphan Drug Designation for branaplam in HD, and a phase IIb trial (NCT05111249) was implemented in 2021. Similarly, PTC Therapeutics leveraged a splicing-focused drug discovery platform to develop compounds mimicking branaplam’s mechanism. These agents successfully lowered HTT mRNA and protein levels in the brain and peripheral tissues (Bhattacharyya et al. 2021). The company launched a phase I trial for PTC518 to assess safety, pharmacokinetics, and pharmacodynamics in healthy individuals, followed by a phase II trial in 2022. These advancements signal growing momentum in small molecule approaches to address the genetic underpinnings of HD (Tabrizi et al. 2022).

## Decoding HD therapies: DNA repair and beyond

### Instability and DNA repair in HD

Frequent DNA is inherently unstable, and over a HD patient’s lifetime the expanded CAG repeat in the HTT gene tends to gain additional repeats, a process known as somatic instability which correlates with earlier disease onset and faster progression (Lee et al. 2019). The SHIELD HD study (NCT04406636) is examining the natural history of somatic instability to inform future therapies targeting repeat expansion. Genome-wide association studies (GWAS) in HD patients highlight DNA repair genes, particularly *FAN1*, as key modifiers of disease onset (Lee et al. 2019). Protective variants of *FAN1* can delay onset by over a year, while deleterious variants accelerate it by more than 5 years (Porro et al. 2021). Mismatch repair pathways, driven by proteins like MSH3 and MLH1, play opposing roles in HD pathogenesis, with some components promoting CAG expansion and others protecting against it. Therapies targeting mismatch repair, such as small-molecule MSH3 inhibitors or ASOs, represent a promising avenue for combating HD at its genetic roots (Moss et al. 2017; Iyer and Pluciennik 2021).

### Pridopidine: a Sigma-1 receptor agonist in HD therapy

Pridopidine, a sigma-1 receptor agonist (Grachev et al. 2021), has shown potential in mitigating HD symptoms. Preclinical studies demonstrated its ability to rescue cell death, restore synaptic plasticity (Smith-Dijak et al. 2019), and improve motor coordination in HD models (Naia et al. 2021). Clinical trials, including HART, MermaiHD, and PRIDE-HD, initially showed limited efficacy on motor outcomes but hinted at improvements in functional capacity, particularly in early HD. These findings led to the ongoing PROOF-HD phase 3 trial (NCT04556656), which aims to further evaluate its impact using functional endpoints. Although its effects on motor symptoms remain modest, pridopidine offers a novel approach by targeting the sigma-1 receptor, with potential implications for disease modification (McGarry et al. 2020).

### Targeting synaptic loss: anti-C1q antibody therapy

Synaptic elimination, a process governed by the classical complement pathway, plays a pivotal role in maintaining neural circuit integrity. This pathway involves synapses tagged with activated C1q protein, which are subsequently cleared by reactive astrocytes. However, dysregulation of this mechanism is implicated in excessive synaptic loss, a hallmark of neurodegenerative conditions such as HD. Studies have identified elevated complement protein levels in HD-affected brains, linking the pathway to disease pathology (Liddelow et al. 2017). In response, Annexon Biosciences has developed ANX-005, an anti-C1q monoclonal antibody designed to inhibit this maladaptive synaptic clearance. ANX-005 is currently being evaluated in a phase 2 open-label trial (NCT04514367) to assess its pharmacokinetics and pharmacodynamics in HD patients, offering a novel therapeutic avenue to preserve synaptic health and potentially mitigate disease progression (García-González et al. 2023).

### Cell therapy aiming for structural and functional restoration

Cell therapy offers a transformative approach to HD by restoring lost neural function and structure. This strategy involves either transplanting cells that replace disease-affected neurons or employing cells that secrete neuroprotective and disease-modifying factors. Initially, efforts focused on replacing degenerated medium spiny neurons (MSNs) using cells from the developing fetal striatum. Preclinical models of HD demonstrated that fetal grafts could reestablish synaptic connections and neural function. Human trials confirmed their safety and feasibility, with preliminary evidence hinting at functional improvement; however, robust efficacy data remain elusive. Given the challenges associated with fetal-derived cells, research has shifted toward MSN-like neurons derived from pluripotent stem cells, which show promising functional outcomes in preclinical studies and potential for clinical application (Björklund and Parmar 2020).

Additionally, non-MSN cell types, including cortical-like neurons and glial progenitor cells, have garnered attention. Glial progenitors, in particular, demonstrated substantial benefits in HD rodent models by improving deficient glial signaling in astrocytes and oligodendrocytes, suggesting their potential role in comprehensive therapeutic strategies for HD (Benraiss et al. 2016).

#### Phosphodiesterase inhibitors

Phosphodiesterase inhibitors (PDEIs) represent a promising therapeutic strategy for HD by targeting the dysregulation of cyclic nucleotides (Cardinale and Fusco 2018; Tyagi et al. 2024). PDEs, which are enzymes that break down cyclic nucleotides, such as cAMP and cGMP, are implicated in the neurodegeneration observed in HD (Bollen and Prickaerts 2012). By inhibiting these enzymes, PDEIs can increase cyclic nucleotide levels, thus impacting various cellular processes and signaling pathways (Lefkowitz 2004). Specifically, the cAMP/PKA/CREB pathway, which is crucial for neuronal survival and plasticity, is impaired in HD, and its modulation through PDEIs has demonstrated neuroprotective effects (Itoh et al. 2004; Gharami et al. 2008; Tyagi et al. 2024). Several PDE families, including PDE1, PDE4, PDE5, and PDE10, have been investigated in the context of HD (Cherry and Davis 1999; Puerta et al. 2009; Puzzo et al. 2009; Cardinale and Fusco 2018).

For instance, vinpocetine, a PDE1 inhibitor, has shown promise in reducing neuroinflammation and improving cognitive function in animal models of HD (Gómez et al. 2014). Rolipram, a PDE4 inhibitor, has demonstrated the ability to increase activated CREB (pCREB) levels and reduce mutant huntingtin inclusions in animal models, although it has been associated with side effects in clinical trials (Giampà et al. 2009a). PDE5 inhibitors like sildenafil and vardenafil have shown potential in ameliorating neurological deficits and upregulating pCREB and BDNF expression in HD models (Puerta et al. 2010). PDE10A inhibitors, such as TP10 and TAK-063, have also exhibited beneficial effects by reducing neuronal loss and increasing lifespan, alongside the upregulation of pCREB and BDNF in animal models (Giampà et al. 2009b, 2010).

These findings indicate that PDEIs can positively impact cortico-striatal transmission by increasing cyclic nucleotide levels in striatal neurons, which can lead to neuroprotection (Colwell and Levine 1995; Sammut et al. 2010). Despite the promising results in animal models, clinical trials with PDEIs have had limited success, highlighting the need for the development of isoform-selective inhibitors to avoid side effects and enhance therapeutic efficacy (Fleischhacker et al. 1992; Cardinale and Fusco 2018). Therefore, the targeted use of PDEIs to potentiate cAMP signaling in the striatum shows a promising approach for future HD therapies (Cardinale and Fusco 2018).

### Artificial intelligence in management of HD

The utilization of artificial intelligence (AI) in treating rare diseases, encompassing HD, is a burgeoning area with considerable promise, although it is still relatively underdeveloped (He et al. 2024). Although AI is predominantly employed in areas like screening, diagnosis, and prognosis for rare diseases (Kumar et al. 2023b; Roman-Naranjo et al. 2023), fewer studies concentrate on therapeutic applications (Vickers 2013). However, AI is being applied in treatment-focused studies to advance drug research, precision medicine, health management, and personalized care (Vickers 2013, Long et al. 2017; Chapron et al. 2021; Foksinska et al. 2022; Kmetzsch et al. 2022; He et al. 2024). AI plays a significant role in drug development, primarily in drug discovery, which involves repurposing existing drugs and pinpointing new drug targets. For instance, machine learning tools have been used to identify potential drugs by correlating disease processes with the effects of drugs (Esmail and Danter 2021; Cong et al. 2022).

One study successfully pinpointed 22 potential drugs for repurposing using a machine learning-based two-stage prediction method (Cong et al. 2022). Ethical considerations such as ensuring the safety and efficacy of AI-developed drugs also need addressing (Naik et al. 2022). In conclusion, while AI offers great potential in improving drug discovery, precision medicine, health management, and personalized care for rare diseases, such as HD, further development is required to address its current limitations (He et al. 2024).

### Immunomodulatory agents

Immunomodulatory agents are being explored as potential treatment for HD, given the significant role of neuroinflammation in the disease pathogenesis and progression (Li et al. 2024). These agents can be classified based on the mechanism of action. Complement inhibitors, like C1q antibodies such as ANX-M1 and ANX005, target the complement system to decrease chronic neuroinflammation. ANX-M1 has demonstrated effectiveness in reversing synaptic issues in HD mouse models (Wilton et al. 2023), while ANX005 has shown safety in Phase 2 clinical trials by lowering neuroinflammation markers. Nonetheless, ANX005 did not produce statistically significant improvements in clinical outcomes (Kumar et al. 2023a). The C1q antibody (ANX005) is expected to enter Phase 3 clinical trials (Field et al. 2024). Semaphorin receptor blockers, such as pepinemab, an antibody that targets SEMA4D, have shown some promise in decreasing neuropathology and cognitive issues in HD mice; however, a Phase 2 trial of pepinemab did not achieve its main objective (Feigin et al. 2022). Kynurenine pathway modulators, such as laquinimod (LAQ), impact the kynurenine pathway and can regulate immune responses in the CNS (Kaye et al. 2016), but it did not show effectiveness in decreasing pro-inflammatory glial activation (Roussakis et al. 2023). In HD, LAQ has shown some promise in improving motor function (Garcia-Miralles et al. 2016; Ellrichmann et al. 2017) and myelin recovery in animal models (Garcia-Miralles et al. 2019; Yin et al. 2020) and in decreasing caudate nucleus atrophy in human trials (Roussakis et al. 2023; Reilmann et al. 2024).

Cytokine modulators focus on specific cytokines involved in inflammation. For instance, the NLRP3 inhibitor MCC950 reduced IL-1β and IL-18, enhancing motor function in HD mice, while overexpression of IL-6 has shown some neuroprotective effects in rat models (Chen et al. 2022). The TNF-α inhibitor Etanercept decreased brain atrophy in HD mice but did not improve motor functions or cognitive deficits (Pido-Lopez et al. 2019). Other immunomodulators include minocycline, which has shown some anti-inflammatory and neuroprotective effects in animal models (Kumar et al. 2013), but did not improve functional outcomes (Smith et al. 2003). In conclusion, while various immunomodulatory agents show potential for treating HD by targeting neuroinflammation, more research is needed to address limitations and improve their effectiveness.

### BDNF mimetics

BDNF mimetics are being explored as a therapeutic approach for HD due to the critical role of BDNF in neuronal survival and function, which are compromised in HD (Kim et al. 2021; Numakawa and Kajihara 2024). These mimetics can be classified into small molecules that directly activate the TrkB receptor via modulating endogenous BDNF levels or compounds that enhance TrkB-mediated signaling indirectly (Sada et al. 2020; Numakawa and Kajihara 2024). One of the most studied small-molecule TrkB agonists is 7,8-dihydroxyflavone (7,8-DHF), which has shown promise in preclinical studies. By binding to TrkB, 7,8-DHF activates PI3K/Akt and MAPK pathways, enhancing neuronal survival and synaptic function in HD models. Another small molecule, LM22A-4, also directly targets and activates TrkB, resulting in activation of downstream pathways like Akt, PLCγ, and CREB. Additionally, LM22A-4 has been shown to reduce mutant huntingtin aggregates, suppress microglial activation, protect striatal neurons, and improve motor function in HD mice (Simmons et al. 2013).

In addition to directly targeting TrkB, some small molecules function by modulating endogenous BDNF levels. For instance, HDAC inhibitors like suberoylanilide hydroxamic acid (SAHA) and CKD-504 have demonstrated neuroprotective effects in HD models, partly by increasing BDNF expression (Mielcarek et al. 2011; Li et al. 2023). Furthermore, natural compounds like curcumin have shown promise due to their ability to upregulate BDNF expression and activate TrkB signaling pathways (Elifani et al. 2019; Kaur et al. 2024). The peptide compound GSB-214 has also demonstrated TrkB agonist activity, promoting neuroprotection (Firouzan et al. 2023). Most of these BDNF mimetics are still in the preclinical stage, with 7,8-DHF (Jiang et al. 2013; García-Díaz Barriga et al. 2017; Ahmed et al. 2021) and LM22A-4 (Yang and Zhu 2022; Numakawa and Kajihara 2024) being the most studied in animal models, while SAHA and CKD-504 are also in preclinical trials (Mielcarek et al. 2011; Li et al. 2023). However, there are no BDNF mimetics are currently in use for the treatment of HD.

## Therapeutic targets/mechanisms of actions of natural products in the management of HD

A variety of treatments derived from natural sources have demonstrated numerous therapeutic benefits for HD in vitro and in vivo models. Recent studies indicate that natural products provide neuroprotection in experimental models mainly by enhancing the antioxidant defense system, reducing oxidative stress, preserving mitochondrial function, offering inflammatory protection, inhibiting apoptosis, and promoting autophagy(Gadade et al. 2024).

Herbal plant components proven to have neuroprotective, immunostimulatory, anti-inflammatory, anti-apoptotic, calcium antagonizing, and antioxidant capabilities have shown promise in the treatment and prevention of HD (Khan et al. 2021). A brief description of various plants and phytochemicals exhibiting anti-HD properties is provided below.

### *Momordica charantia*

*Momordica charantia* L., is frequently referred to as karela, bitter gourd, and/or bitter melon. The impact of bitter gourd on cognitive skills was assessed in scopolamine-induced amnesia in rats utilizing the plus maze test, passive avoidance, and motor activity paradigms. Pre-treatment with bitter gourd (150, 300, and 600 mg/kg, orally) enhanced learning and memory metrics in a concentration-dependent manner (Joshi et al. 2017).

The neuroprotective efficacy of the predominant phytoconstituent of *M. charantia* L., charantin (1 mg/1 mL), was assessed through in vitro research utilizing SH-SY5Y neuroblastoma cell lines, wherein neuronal injury was induced by 1-methyl 4-phenylpyridinium (MPP) and tunicamycin (a bacterial toxin). The results demonstrated that pre-treatment with charantin influenced cell survival in a time-dependent manner, which is ascribed to the free radical scavenging ability of charantin (Tamilanban 2018).

### *Phoenix dactylifera*

*Phoenix dactylifera* Linn, belonging to the Arecaceae family, is commonly referred to as the date palm. Date palm fruits possess significant nutritional value and are abundant in carotenoids, sterols, flavonoids, lignans, and phenolic acids, all of which function as effective antioxidants. Recent reports indicate neuroprotective effects in a 3-NP induced HD in vitro model. The ethanolic extract of date palm fruits improved oxidative stress-induced mitochondrial dysfunction, as indicated by the restoration of intracellular ATP production. Date palm fruits’ antioxidant capabilities were thought to be responsible for the decrease in ROS production and the increase in endogenous GSH and SOD levels (Tamilanban 2018).

### *Panax ginseng*

Ginseng extract enhances neurological and psychological symptoms, as well as cognitive functions, in healthy individuals. An in vitro HD assay utilizing MSN cultures to investigate the neuroprotective potential of ginseng total saponins demonstrates that Rb1, Rg5, and Rc are associated with the ability to mitigate glutamate-induced Ca2 + responses. Ginseng positively influences psychological performance due to its effects on the hippocampal region of the brain. The inhibition of Ca2 + entry via glutamate receptors by ginsenosides Rb1 and Rg3 provides protection to cortical neurons against glutamate-induced cellular damage. Furthermore, it reduces lipid peroxidation, lowers neuronal excitotoxicity, stabilizes ATP levels in cells, maintains neuronal structural integrity, and improves cognitive performance (Rai et al. 2017; Khan et al. 2020).

### *Bacopa monnieri*

The pharmacological properties of *B. monnieri* are attributed to the presence of numerous active constituents, including saponin, alkaloids, sterols, and flavonoids. This plant has the potential to be a memory enhancer, anti-inflammatory, analgesic, hepatoprotective, and antipyretic, as well as an antidepressant agent and free radical scavenger for neuropharmacological disorders such as insomnia(Abdul Manap et al. 2019).

The mechanisms involved, such as metal ions complex chelation and the enhancement of antioxidant defense enzymes, contribute to the neuroprotective and memory-boosting effects of *B. monnieri* extracts. An ethanolic extract of *B. monnieri* inactivates 3-NP induced mitochondrial dysfunction by modifying the antioxidant mechanism (Fatima et al. 2022).

3-NP inhibits the succinate dehydrogenase enzyme (SDH) and affects the electron transport chain complex II–III. It also decreases levels of free fatty acids, ROS, and malondialdehyde (MDA). The consumption of *B. monnieri*’s leaf powder has been documented to lower baseline levels of various oxidative markers and enhance thiol-related antioxidant molecules, as well as the activity of antioxidant enzymes. The dietary *B. monnieri* supplements provide significant protection against oxidative impairment in the brain and offer a defensive effect against neuronal dysfunction caused by stress. Therefore, *B. monnieri* may provide significant advantages in the treatment of HD (Vishwas et al. 2021).

### *Xylaria species*

Xyloketal B is an extract derived from the marine mangrove fungus of the Xylaria species. Xyloketal B has demonstrated significant neuroprotection in many models associated with neuronal dysfunction. Six derivatives of xyloketal B were assessed in a Caenorhabditis worm model of HD to identify effective neuroprotective agents; all six compounds exhibited a protective effect. The aromatic core structure of Xyloketal B contains distinctive bicyclic acetal moieties that can be readily modified to enhance and expand its action. Additionally, certain xyloketal derivatives are capable of forming hydrogen bonds. Xyloketal binds to mutant htt proteins and impedes the htt aggregation process, hence decelerating the advancement of HD (Gong et al. 2022).

### *Luehea divaricata*

Alkaloids, anthocyanidins, polysaccharides, carotenoids, and some fixed oils are present in the crude extract of *Luehea divaricata*, while mart leaves are said to be abundant in flavonoids, saponins, mucilage, and tannins. In the rat model (Courtes et al. 2015) examined the neuroprotective effect of *Luehea divaricata* extract against HD-associated behavioral and biochemical alterations brought on by 3-NP. Remarkably, the aqueous extract of *Luehea divaricata* (500 and 1000 mg/kg, ir) seems to correct the motor and behavioral abnormalities, enhancing locomotor and rotarod performance. Furthermore, the biochemical change was lessened in the striatum and cortex, which corresponded to a decrease in oxidative stress and lipid peroxidation. This suggests that the plant extract’s potent antioxidant activity protected against HD in vivo.

### *Sesamum indicum*

The plant comprises numerous active ingredients, namely acteoside, pedaliin, luteolin, cistanoside, sesamoside, and sesamol. Sesamol is the most active phenolic chemical derived from *Sesamum indicum* L. The protective effect of sesamol against 3-NP-induced cognitive impairment (neurotoxicity) and oxidative damage in the striatum, cortex, and hippocampus regions of the brain was assessed in male Wistar rats. Pre-treatment with sesamol (10 mg/kg, i.p.) markedly enhanced body weight, locomotor activity, and diminished oxidative damage by decreasing lipid peroxidation (LPO) while elevating catalase (CAT) and superoxide dismutase (SOD) levels in rat brains, thereby presenting a potentially viable strategy for the management of HD (Lum et al. 2021).

### *Triptergiumwilfordi (TW)*

Celastrol and quinone methide triterpenoid are the principal active compounds in TW that account for its therapeutic efficacy. However, celastrol is the primary active component of TWHF accountable for its biological activity (Li and Hao 2019). Mice were administered celastrol (3 mg/kg, i.p.) in order to determine its anti-Huntington impact using the 3-NP model of HD. Celastrol has the potential to be a neuroprotective drug for the treatment of HD since it reduced the volume of striatal lesions by reducing NF-kB and TNF-α (Singh et al. 2022).

### *Zingiber officinale (ginger)*

The presence of many phytochemicals, including 6,8.10-gingerol, 6,8,10-shogaol, and zingiberene, is responsible for ginger’s health advantages (Roli et al. 2020). In male Wistar rats, (Akila and Shaji 2022) tested the anti-Huntington efficacy of ginger-loaded chitosan nanoparticles (NPs) against 3-NP-induced HD. Neuroprotection was demonstrated in both free ginger (10 mg/kg, i.p.) and chitosan-loaded nanoparticle formulation (10 mg/kg, i.p.), attributed to the antioxidant activity of 6-shogaols through the suppression of microglial cells during neuronal injury.

### *Punica granatum*

Punica granatum exhibit antioxidative qualities in PC-12 cells and the brain, and has the ability to minimize cellular oxidative stress, reverse the decline in antioxidants, and sequester free radicals. In order to counteract the excess ROS in the brain, Nrf2 activation triggers the endogenous antioxidative defence system, which in turn causes the overexpression of enzymatic (SOD, CAT, and GPx) and nonenzymatic antioxidants (GSH) (Al-Sabahi et al. 2017; Danduga et al. 2018).

### *Ginkgo biloba*

*Ginkgo biloba*, a traditional medicinal plant rich in flavonoids and terpenoids, has been widely studied for its neuroprotective properties. Its potential role in the management of HD is attributed to its antioxidant, anti-inflammatory, and mitochondrial-stabilizing effects. Additionally, its ability to enhance cerebral blood flow and synaptic plasticity has drawn attention in the context of HD and other neurodegenerative disorders. A 2022 review highlighted the potential of natural products, including *Ginkgo biloba* extract (GBE), in managing HD by modulating oxidative stress and related molecular pathways. The findings emphasized that compounds like GBE can mitigate oxidative damage, reduce neuronal apoptosis, and modulate signaling pathways implicated in HD pathogenesis, offering a promising avenue for therapeutic development (Irfan et al. 2022). Additionally, a 2018 study demonstrated that Ginkgolide B, a component of GBE, promotes neuronal differentiation through the Wnt/β-catenin signaling pathway, which is crucial for cell survival and neuroprotection. The researchers found that Ginkgolide B enhances the expression of neuronal markers and supports the maturation of neural stem cells, suggesting its potential in promoting neurogenesis and repairing neural damage (Li et al. 2018). Mechanistic studies in HD models indicate that *Ginkgo biloba* enhances mitochondrial respiration and reduces neuronal apoptosis, potentially contributing to slowing disease progression. These findings align with its broader neuroprotective potential in neurodegenerative conditions (Gupta et al. 2022).

### *Withaniasomnifera (Ashwagandha)*

*Withania somnifera*, known as Ashwagandha, has been extensively studied for its neuroprotective and anti-inflammatory properties (Sharma 2023). The plant’s bioactive compounds, withanolides, exhibit anti-aggregative properties against mutant huntingtin protein, along with enhancing synaptic plasticity and reducing excitotoxicity (Joshi et al. 2021). Ashwagandha is also known for its adaptogenic properties, which contribute to stress reduction, a crucial factor in neurodegenerative diseases (Speers et al. 2021). A 2023 review discussed the neuroprotective role of *W. somnifera* in several neurodegenerative conditions. The study highlighted that *W. somnifera* has demonstrated the ability to reverse amyloid-induced toxicity, inhibit acetylcholinesterase activity, and increase the expression of neuroprotective proteins. In the context of HD, *W. somnifera* has been shown to reduce neuronal damage and oxidative stress. These neuroprotective properties are attributed to the plant’s antioxidant and anti-inflammatory effects (Dar et al. 2023). Moreover, a 2020 study emphasized the neuroprotective properties of *W. somnifera* extract and its active constituents in neurodegenerative diseases, including HD. The protective effects of *W. somnifera* were accomplished by restoring mitochondrial and endothelial function, and mitigating apoptosis, inflammation, and oxidative stress mechanisms (Dar and Ahmad 2020). These findings suggest that *W. somnifera* may offer therapeutic benefits for HD by mitigating oxidative damage and enhancing neuronal survival mechanisms.

## Natural compounds interacting with one or more targets and evidence supporting their use in the management of HD

**Fig. 3:**
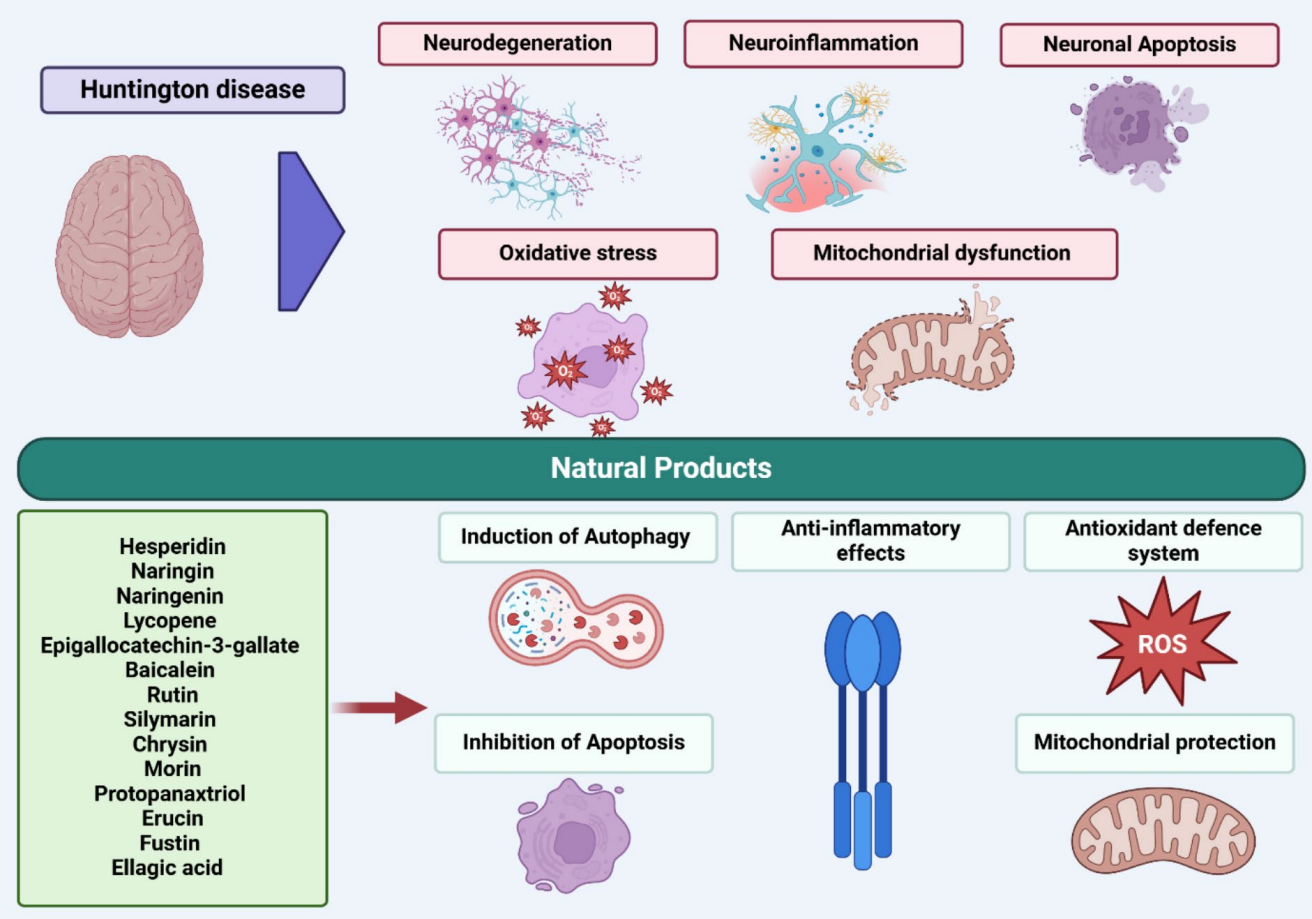
Neuroprotective role of natural products in the management of Huntington’s disease (HD)

Natural products offer potential therapeutic benefits through mechanisms such as antioxidant activity, anti-inflammatory effects, and the preservation of mitochondrial function (Aly et al. 2023, 2024a, 2024b; Abdelazim et al. 2024). HD is a neurodegenerative condition marked by advancing physical impairment, mental health issues, and cognitive deterioration. Although there is no cure for HD, natural compounds have demonstrated potential in preclinical research by providing neuroprotective effects via various mechanisms. Antioxidant action by mitigating oxidative damage through the elimination of free radicals and the enhancement of the activity of antioxidant enzymes (Cusumano et al. 2024; Zengin et al. 2024). Anti-inflammatory properties by inhibiting inflammatory pathways that contribute to neurodegeneration (Aly et al. 2022). Preserving mitochondrial function to prevent energy deficits in neurons. Anti-apoptosis effects by preventing programmed cell death of neurons and autophagy induction. Several natural products have demonstrated the ability to manage HD, with specific examples detailed in the subsequent section and represented in Table 1 and Fig. 3.

**Table 1:** Summary of the role of natural compounds on the management of Huntington’s disease (HD) in different animal models

Natural compound	Class of compound	Dose	Model	Effects	Reference
Hesperidin	Flavonoid	100 mg/kg	3-Nitropropionic acid (3-NP) induced neurotoxicity	↑ Catalase activity ↓ Abnormalities induced by 3-NP Prevented changes of locomotor activity or PPI response Antioxidant, anti-inflammatory, and anti-apoptotic properties	(Menze et al. 2012; Chien et al. 2018)
Hesperidin and naringin	Flavonoid	(50 mg/kg)	3-NP induced neurotoxicity	↓Oxidative stress Restored antioxidant defenses Mitigation of mitochondrial enzyme depletion	(Kumar and Kumar 2010)
Naringin	Flavonoid	40 and 80 mg/kg	Quinolinic acid (QA)-induced HD	↓Oxidative stress markers ↓Neuroinflammation ↓Caspase-3 ↓Apoptosis Modulated Bax/Bcl-2 ratio Enhanced mitochondrial complex (I-IV) activity	(Cui et al. 2018)
		80 mg/kg	3-NP induced neurotoxicity	Activates Nrf2 ↑Antioxidant Enzymes ↓Inflammation	(Gopinath and Sudhandiran 2012)
Naringenin	Flavonoid	50 mg/kg	3-NP induced neurotoxicity	Alleviate symptoms related to motor dysfunction and cognitive decline ↓Neuronal cell death and ↓The expression of glial fibrillary acidic protein (GFAP)	(Salman et al. 2022)
Hesperidin and minocycline	Flavonoid and antibiotic	Hesperidin (100 mg/kg, p.o.) and minocycline (25 mg/kg, p.o.)	QA-induced HD	Improved memory performance ↓Oxidative stress markers ↑Superoxide dismutase ↑Glutathione ↓TNF-α ↓Neuroinflammation ↓Caspase-3 ↓Apoptosis Mitochondrial protection	(Kumar et al. 2013)
Lycopene and epigallocatechin-3-gallate (EGCG)	Polyphenolic and carotenoid	Lycopene (2.5, 5 and 10 mg/kg) EGCG (10, 20 and 40 mg/kg)	3-NP induced neurotoxicity	Improves memory Restores glutathione levels Prevent mitochondrial oxidative stress and dysfunction	(Kumar and Kumar 2009)
Baicalein	Flavonoid	30 mg/kg	QA-induced HD	Improved cognitive functions ↑Memory & learning Restore BDNF and GDNF levels ↓Oxidative stress Restored mitochondrial function ↓Neuroinflammation ↓Inflammatory mediators	(Purushothaman and Sumathi 2022)
Rutin	Flavonoid	9.16–73.26 mg rutin/mL agar	*Caenorhabditis elegans* model	Prevention of neurodegeneration Maintenance of cellular homeostasis Prevention of neuronal death ↓Oxidative stress ↑Autophagy	(Cordeiro et al. 2020)
Silymarin	Flavonolignans	100 mg/Kg	3-NP induced neurotoxicity	Mitigate the motor symptoms antioxidant, anti-inflammatory, and anti-apoptotic properties	(Aliaghaei and Meftahi 2024)
Chrysin	Flavonoid	50 mg/Kg	3-NP induced neurotoxicity	Improves mitochondrial function ↓Lipid peroxidation, nitrite, and protein carbonyls ↑Superoxide dismutase, catalase, and reduced glutathione	(Thangarajan et al. 2016)
Morin	Flavonoid	10 and 5 mg/Kg	3-NP induced neurotoxicity	Modulates the glutamate/calpain axis Modulating Kidins220 protein Promotes neuroprotection and recovery from neurotoxicity	(Mohamed et al. 2023)
Protopanaxtriol	Steroid glycoside	5, 10, and 20 mg/Kg	3-NP induced neurotoxicity	↓Reactive oxygen species (ROS) ↑Nuclear translocation of Nrf2 ↓ Heme oxygenase-1 (HO-1) and NAD(P)H quinone oxidase 1 (NQO1) Modulate heat shock protein 70 (Hsp70) expression,	(Gao et al. 2015)
Erucin	Isothiocyanate	100 μM and 200 μM	*Caenorhabditis elegans*	↓Formation of polyglutamine aggregates in the ventral nerve cord Activation of aak-2/AMPK Pathway Activation of daf-16/FOXO Transcription Factor Alleviates motor and cognitive impairments	(Balducci et al. 2024)
Quinic acid	Cyclitol and cyclohexanecarboxylic acid	50, 100 and 200 μg/mL	*Caenorhabditis elegans*	↓ Formation of huntingtin aggregates ↓ Oxidative stress markers ↓ Apoptosis Activation of SKN-1/Nrf2 pathway	(El Din and Thabit 2024)
Fustin	Flavonoid	50 and 100 mg/Kg	3-NP induced neurotoxicity	↑GSH, SOD, and CAT levels ↓MDA levels Modulates GABA and glutamate levels ↑BDNF activity	(Bin-Jumah et al. 2022)
Ellagic acid	Phenolic acid	25, 50, and 100 mg/kg	3-NP induced neurotoxicity	Improved cognitive functions Restored succinate dehydrogenase activity Maintenance of mitochondrial function ↓ Markers of oxidative stress and nitrosative stress in the brain	(Sharma et al. 2021)

Pretreatment with hesperidin (100 mg/kg) before exposure to 3-nitropropionic acid (3-NP) has been shown to prevent changes in locomotor activity and prepulse inhibition (PPI) response in rats. This suggests that hesperidin may protect against the neurobehavioral impairments caused by 3-NP. While hesperidin generally acts as an antioxidant, in the context of 3-NP-induced neurotoxicity, it slightly increased MDA levels in the cortex, striatum, and hippocampus by about 10%. However, it reduced catalase activity in these regions by 22%, 20%, and 5%, respectively. This mixed effect on oxidative stress markers indicates a complex interaction between hesperidin and 3-NP-induced oxidative damage (Menze et al. 2012).

Hesperidin and naringin pretreatment significantly attenuated the behavioral alterations caused by 3-NP, such as reduced locomotor activity, body weight, and grip strength. Both compounds reduced oxidative stress by mitigating the depletion of antioxidant defences and mitochondrial complex enzymes (I, II, and IV) in the striatum. The study suggests that hesperidin and naringin may act through a nitric oxide mechanism. L-NAME, a non-selective NOS inhibitor, potentiated their protective effects, while L-arginine, a nitric oxide precursor, attenuated these effects (Kumar and Kumar 2010).

The neuroprotective properties of naringin, a flavone glycoside, against QA-induced neurodegeneration incorporate multiple mechanisms, including modulation of oxidative-nitrosative stress, neuroinflammation, and apoptosis. Naringin reduces oxidative-nitrosative stress by modulating ROS and reactive nitrogen species (RNS), which are elevated in QA-induced neurotoxicity. It decreases neuroinflammatory markers, contributing to its neuroprotective effects. Also, it modulates apoptotic markers, including Bax/Bcl-2 ratio and caspase-3 activity, to prevent neuronal death. Moreover, naringin may exert its neuroprotective effects by activating peroxisome proliferator-activated receptor gamma (PPAR-γ), which plays a role in anti-inflammatory and antioxidant pathways (Cui et al. 2018).

Another study has shown that naringin can influence oxidative stress and inflammation in neurodegenerative incidents, namely in models of neurodegeneration generated by 3-nitropropionic acid. Naringin triggers Nrf2, a vital transcription factor that plays a key role in cellular defense systems against oxidative stress. By activating Nrf2, naringin increases the expression of antioxidant enzymes such as NAD(P)H:quinone oxidoreductase-1, heme oxygenase-1, glutathione S-transferase P1, and gamma-glutamylcysteine ligase. This leads to reduced concentrations of oxidative stress indicators such as hydroxyl radicals and hydroperoxides. The activation of Nrf2 by naringin contributes to neuroprotection by mitigating oxidative stress and inflammation, which are key factors in neurodegenerative diseases. This makes naringin a potential therapeutic agent for conditions like HD, where oxidative stress plays a significant role (Gopinath and Sudhandiran 2012).

Naringenin co-treatment reduces behavioral alterations associated with 3-NP-induced neurotoxicity, suggesting its potential to alleviate symptoms related to motor dysfunction and cognitive decline in neurodegenerative diseases like HD. It diminishes neuronal cell death and mitigates the expression of glial fibrillary acidic protein (GFAP), a biomarker of astrocyte activation, which represents neuroinflammation. Besides, it showed modulation of neurotransmitter systems involved in mood regulation and motor function (Salman et al. 2022).

In another investigation, the administration of QA markedly disrupted locomotor activity and balance in rats, an effect that was mitigated by prolonged treatment with hesperidin (100 mg/kg) and minocycline (25 mg/kg). The treatment enhanced memory performance relative to QA-treated controls. Hesperidin and minocycline administration decreased oxidative stress indicators and increased SOD and GSH levels. The combined therapy lowered pro-inflammatory cytokines such as TNF-α and caspase-3, signifying less neuroinflammation and increased expression of brain-derived neurotrophic factor (BDNF), essential for neuronal survival. Furthermore, combination therapy facilitated mitochondrial protection by diminishing apoptosis via the modulation of BDNF and caspase-3 levels (Kumar et al. 2013).

Both lycopene and epigallocatechin-3-gallate (EGCG) have been shown to mitigate the neurotoxic effects induced by 3-NP by improving memory performance and restoring glutathione levels, which are crucial for antioxidant defence. Lycopene has been demonstrated to inhibit mitochondrial oxidative stress and dysfunction induced by 3-NP. By reducing oxidative damage and improving glutathione levels, both lycopene and EGCG contribute to enhanced cognitive function in rats exposed to 3-NP (Kumar and Kumar 2009).

The study on baicalein effects against QA-induced Huntington’s-like disease in rat striatum provides insights into its potential neuroprotective role. It may help improve cognitive impairments caused by QA, potentially enhancing memory and learning abilities. Furthermore, the study indicates that baicalein restores the levels of brain neurotrophins, including BDNF and GDNF, which are essential for neuronal survival and mental functioning. It probably diminishes oxidative stress and inflammation, which are critical contributors to neurotoxicity induced by QA (Purushothaman and Sumathi 2022).

Rutin’s protective effects are partly attributed to its modulation of the insulin/IGF1 signalling pathway. This pathway plays a crucial role in regulating metabolism, growth, and longevity, and its dysregulation is linked to neurodegenerative diseases. Rutin promotes autophagy, a cellular mechanism that facilitates the destruction and recycling of impaired cellular constituents. This is crucial for sustaining cellular homeostasis and averting neurodegeneration. Furthermore, rutin diminishes the aggregation of polyglutamine proteins, which are harmful in HD and contribute to neuronal apoptosis. It also demonstrates potent antioxidant capabilities, alleviating oxidative stress and safeguarding against cellular damage in the *Caenorhabditis elegans* model (Cordeiro et al. 2020).

Erucin, a natural isothiocyanate found in certain vegetables like rocket salad, has been shown to prevent polyglutamine-induced toxicity in *Caenorhabditis elegans* by activating specific signalling pathways. Erucin reduces the formation of polyglutamine aggregates in the ventral nerve cord of *C. elegans*, which are associated with neurodegenerative diseases like HD. The protective effects of erucin are mediated by the activation of the *aak-2*/AMPK (adenosine monophosphate-activated protein kinase) pathway and the *daf-16*/FOXO (forkhead box O) transcription factor. AMPK activation enhances energy homeostasis, while FOXO promotes transcriptional regulation of genes involved in stress resistance and longevity. Also, it restores neuronal function and reduces muscular toxicity in *C. elegans* expressing polyglutamine expansions. The reduction in polyglutamine aggregation by erucin requires the catalytic function of AMPK and the transcriptional activity of FOXO, highlighting the importance of these pathways in mitigating neurodegenerative processes (Balducci et al. 2024).

Quinic acid treatment significantly improved locomotion and overall health of the *C. elegans* models, suggesting enhanced motor function. The compound effectively reduced the formation of huntingtin aggregates, which are toxic protein clumps associated with HD pathology. Quinic acid exhibited antioxidant properties, reducing oxidative stress markers within the nematodes through activation of the SKN-1/Nrf2 pathway. The study proposed that quinic acid may exert its protective effects through modulation of cellular pathways related to stress response and apoptosis (El Din and Thabit 2024).

In a recent study, Silymarin treatment improved motor function in rats with HD, suggesting its potential to mitigate the motor symptoms associated with the disease. By averting neuroinflammation-induced cell death, silymarin may help preserve neuronal integrity. The neuroprotective benefits of silymarin are ascribed to its antioxidant, anti-inflammatory, and anti-apoptotic characteristics. These mechanisms mitigate oxidative stress and inflammation, which are significant factors in neurodegeneration in HD (Aliaghaei and Meftahi 2024).

Chrysin, a bioactive flavonoid, has been shown to exert neuroprotective effects towards 3-NP induced neurodegeneration in male Wistar rats. Chrysin improves mitochondrial function by enhancing the activity of mitochondrial complexes, which are crucial for energy production and cellular survival. It decreases oxidative stress markers such as lipid peroxidation, nitrite, and protein carbonyls, while increasing antioxidant defenses like SOD, CAT, and GSH. Moreover, chrysin upregulates the anti-apoptotic Bcl-2 gene and downregulates the pro-apoptotic Bax and Bad genes, thereby preventing apoptosis and promoting neuronal survival. Furthermore, it improves behavioral performances in rats, suggesting its potential to mitigate the behavioral despair associated with neurodegenerative conditions (Thangarajan et al. 2016; Haider et al. 2022).

Morin modulates the glutamate/calpain axis, which is crucial in neurodegenerative processes in nitropropionic acid (3-NP)-induced cortical neurotoxicity model. Morin also affects Kidins220, a protein involved in neuronal survival and synaptic plasticity. Modulating Kidins220 can enhance neuronal resilience against neurotoxic insults. Besides, morin activates the brain-derived neurotrophic factor (BDNF)/tropomyosin receptor kinase B (TrkB)/protein kinase B (AKT)/cAMP response element-binding protein (CREB) signalling pathway. This pathway is essential for neuronal survival, growth, and synaptic plasticity. Activation of this pathway promotes neuroprotection and recovery from neurotoxicity (Mohamed et al. 2023).

Protopanaxtriol (Ppt), a steroid compound derived from *Panax ginseng*, has demonstrated protective effects against oxidative stress generated by 3-NP in a rat model of HD. Ppt mitigates 3-NP-induced oxidative stress by reducing ROS production and restoring antioxidant enzyme activity in the striatum. Ppt promotes the nuclear translocation of Nrf2. This results in elevated levels of downstream antioxidant enzymes, including heme oxygenase-1 and NAD(P)H quinone oxidase 1 (NQO1). Ppt’s neuroprotective properties have been attributed to its capacity to diminish neuronal injury and regulate the expression of heat shock protein 70 (Hsp70), a marker of cellular stress response (Gao et al. 2015).

Fustin downregulates oxidative stress by enhancing antioxidant defenses, such as increasing levels of GSH, SOD, and CAT, while decreasing MDA levels. This helps mitigate the oxidative damage associated with 3-NP-induced neurotoxicity. It modulates neurotransmitter levels, particularly GABA and glutamate, which are crucial for maintaining neuronal balance and function. This modulation helps restore normal neurotransmitter activity disrupted by 3-NP. Besides, fustin enhances BDNF activity, which is essential for neuronal survival, growth, and synaptic plasticity. Increased BDNF activity contributes to neuroprotection and recovery from neurotoxicity. Regarding behavioral improvements, fustin improves behavioral outcomes in rats, as evidenced by enhanced performance in beam walk, rotarod, and grip strength tests. These improvements suggest that fustin can mitigate motor dysfunction and cognitive decline associated with HD (Bin-Jumah et al. 2022).

Ellagic acid (EA) administration markedly enhanced cognitive functioning, as evaluated by novel object recognition and raised plus maze assessments, signifying improved memory and less anxiety-like behavior. It effectively protected against mitochondrial dysfunction caused by 3-NP as ellagic acid pre-treatment restored succinate dehydrogenase activity in 3-NP treated rats, indicating the maintenance of mitochondrial function. Also, it demonstrated significant antioxidant effects, reducing markers of oxidative stress and nitrosative stress in the brain. The study suggests that EA may exert its protective effects through mechanisms involving free radical scavenging, modulation of cell signalling pathways, and reduction of inflammation (Sharma et al. 2021).

In an in vitro study aimed to elucidate the mechanisms by which vanillin protects against rotenone-induced neurotoxicity, SH-SY5Y neuroblastoma cells were treated with varying concentrations of rotenone (5–200 nM) to induce neurotoxicity. Vanillin was administered before rotenone exposure to assess its protective effects on cell viability, mitochondrial function, and oxidative stress levels. Vanillin treatment significantly improved cell viability in rotenone-treated SH-SY5Y cells. The compound preserved mitochondrial membrane potential and reduced the generation of ROS, indicating its role in mitigating oxidative stress. Also, vanillin treatment altered the expression patterns of apoptotic markers, including the upregulation of anti-apoptotic proteins and the downregulation of pro-apoptotic proteins. The study highlighted that vanillin inhibited pathways associated with cell death, including p38 and JNK-MAPK signalling pathways (Dhanalakshmi et al. 2015).

The previous studies underscore the potential of natural compounds in developing strategies for treating neurodegenerative diseases such as HD. Further studies are needed to explore the clinical applicability of these findings and the underlying mechanisms through which natural products exert their effects.

## Conclusions

In this review, we aimed to present a comprehensive overview of current research trends on the anti-HD effects of natural products. Due to the complexity of HD, a single compound acting on one or a few targets cannot govern it. Because herbal extracts contain a variety of compounds, it is possible that synergistic interaction between phytochemicals improves their efficacy and allows them to operate on many targets to control complicated disorders. However, a number of factors may have contributed to a slowdown in natural products-based research and development. These could include inadequate quality control in herbal products, as well as low repeatability of bioactivity, bioavailability, and biotransformation of active compounds. There are also intricate challenges concerning natural compound metabolism and dose–response interactions, as well as a lack of information on clinical results from numerous research. In-depth study is still required to find and refine novel plant-based bioactive compounds, to assess the therapeutic efficiency of these compounds, and to clarify the underlying mechanism of herbal plants action as useful and safe treatments for the future.

## Data Availability

All source data for this work (or generated in this study) are available upon reasonable request.
